# Single nucleotide polymorphisms in CIDEC gene are associated with metabolic syndrome components risks and antihypertensive drug efficacy

**DOI:** 10.18632/oncotarget.16078

**Published:** 2017-03-10

**Authors:** Hui Wang, Yun Ti, Jin-Bo Zhang, Jie Peng, Hui-Min Zhou, Ming Zhong, Yan-Qiu Xing, Yun Zhang, Wei Zhang, Zhi-Hao Wang

**Affiliations:** ^1^ The Key Laboratory of Cardiovascular Remodeling and Function Research, Chinese Ministry of Education and Chinese Ministry of Health, The State and Shandong Province Joint Key Laboratory of Translational Cardiovascular Medicine, Department of Cardiology, Qilu Hospital of Shandong University, Jinan, Shandong, 250012, P.R. China; ^2^ Weihai Center for Diseases Control and Prevention, Weihai, Shandong, 264200, P.R. China; ^3^ Department of Geriatrics, Qilu Hospital of Shandong University, Jinan, Shandong, 250012, P.R. China

**Keywords:** metabolic syndrome, single nucleotide polymorphisms, risk factor, pharmacogenomics, drug treatment

## Abstract

The association of single nucleotide polymorphisms rs1053239 and rs2479 of cell death-inducing DFFA-like effector c with the risk of metabolic syndrome and its components, and with the efficacy and cost-effectiveness of antihypertensive drugs was investigated. Totally 1064 subjects with metabolic syndrome and 1099 controls of Chinese Han nationality were recruited. Clinical assessment was conducted with medication records collected at baseline and during 5-year follow-up. Carriers of rs2479 A allele were at higher risk to develop elevated fasting glucose than non-carriers (*P* = 0.004). A allele at rs2479 were associated with a 5-year aggravation of blood triglyceride (*P* < 0.001) and diastolic blood pressure (*P* = 0.003), and C allele at rs1053239 with the exacerbation of systolic (*P* < 0.001) and diastolic blood pressure (*P* = 0.001). Moreover, efficacy and cost-effectiveness of angiotensin II-targeted drugs were higher in subjects with rs2479 A allele or rs1053239 C allele. These findings suggest that carriers of rs2479 A allele are predisposed to the development of increased fasting glucose, and the progressive elevation of blood triglyceride. Individuals with A allele at rs2479 or C allele at rs1053239 are more susceptible to a rapid progression of blood pressure, and benefit more from angiotensin II-targeted therapy.

## INTRODUCTION

The prevalence of the metabolic syndrome (MS) is globally rising and in China, it was 9.8% in men and 17.8% in women in 2000–2001 [1]. MS and its components are associated with increased risk of cardiovascular disease [2] and oncogenesis [3], and cast a heavy economic burden on public medical budgets [4]. Insulin resistance is considered as the trigger of MS, and dysfunction of glucose and lipid metabolism as the core manifestation [5]. The etiology of MS is complicated involving the interaction of multiple genes and environmental factors, the mechanism of which remains elusive. Factors that interfere with glucose or lipid homeostasis are proposed to potentially confer an influence on the development, progression, and intervention effects of MS, or its individual components.

The lipid droplet-associated protein cell death-inducing DFFA-like effector c (*CIDEC*) has gained increasing attention as a vital regulator in lipid and glucose metabolism and insulin sensitivity. It was implicated to facilitate adipocytes differentiation [6] and repress lipid mobilization [7–9]. Systemic [10] or adipose tissue-specific ablation [11] of *CIDEC* in mice substantially enhanced lipolysis, diminished adipose tissue mass, and triggered hepatic lipid deposition, dyslipidemia and systemic insulin resistance. Therefore, *CIDEC* might act as an underlying node tuning systemic metabolic fluctuation, and could serve as a candidate gene for the development of MS and its components.

Human data showed the amount of *CIDEC* mRNA in white adipose tissue was decreased in obesity [12, 13], and positively correlated with insulin sensitivity [12–14]. In addition, a case with familial partial lipodystrophy 5 was reported to harbor a nonsense mutation at the sixth exon of *CIDEC* [15]. To date, however, there has been a lack of population-based association analysis on *CIDEC* genetic variation with the risk of MS or its components, or with the longitudinal changes of MS components in follow-up study.

Diversity exists in drug response among population, and genetic variation has been broadly conceived to be largely responsible for the inter-individual variability [16]. Based on this, pharmacogenetics offers the opportunity for precision medicine to improve drug efficacy and guide cost-effective medical decision [17, 18]. The pathogenesis of MS involves multiple proteins in interlaced molecular pathways, which might serve as targets for the pharmacological action of drugs correcting metabolic disturbance. As a role of *CIDEC* was proposed in the development of MS, it deserved exploration whether *CIDEC* genetic variation might affect the efficacy of medication on MS or its components. Moreover, economic expense should be taken into account into drug efficacy evaluation to weigh the cost and benefit of medication therapy [18].

Herein, we sought to explore the single nucleotide polymorphisms (SNPs) of *CIDEC* at rs1053239 and rs2479 in relation to the risk of MS and its components in case-control and follow-up studies. Furthermore, the impact of *CIDEC* genetic variation on the efficacy and cost-effectiveness of antihypertensive agents was assessed to facilitate medication choice in hypertension treatment.

## RESULTS

### Baseline characteristics of the study population

Anthropometric and biological characteristics of subjects in the initial of the investigation were summarized in Table 1. Participants were sex- and age- matched in Control and MS groups. The overall minor allele frequency (MAF) was 0.409 (0.374 for Han Chinese in Beijing, CHB; the Ensembl Database, http://www.ensembl.org/index.html) for rs1053239 and 0.261 (0.252 for CHB; the Ensembl Database) for rs2479. Genotype distribution in Control, MS and total subjects were in Hardy-Weinberg equilibrium, respectively (Supplementary Table 1).

**Table 1:** Baseline clinical and biological characteristics of participants in Control and MS group

	Control	MS
	(*n* = 1099)	(*n* = 1064)
Male (%)	43.1	40.8
Age (years)	49.57 ± 11.63	49.83 ± 8.67
BMI (kg/m^2^)	21.98 ± 2.55	27.39 ± 3.13 ^a^
WC (cm)	74.93 ± 6.13	90.91 ± 7.70 ^a^
SBP (mmHg)	115.76 ± 8.41	148.51 ± 17.84^a^
DBP (mmHg)	73.56 ± 6.11	90.64 ± 9.60 ^a^
TG (mmol/L)	0.91 ± 0.31	2.13 ± 1.07 ^a^
TC (mmol/L)	4.11 ± 0.55	4.60 ± 1.03 ^a^
HDL (mmol/L)	1.58 ± 0.32	1.46 ± 0.44 ^a^
LDL (mmol/L)	2.53 ± 0.54	3.13 ± 0.73 ^a^
FPG (mmol/L)	4.55 ± 0.50	5.77 ± 1.61 ^a^
Smoking (%)	21.6	13.3 ^a^
Alcohol intake (%)	17.6	27.0 ^a^

Data are mean ± SD for continuous variables, or proportions for categorical variables. ^a^ *P* < 0.001 vs. Control. MS for metabolic syndrome; BMI, body mass index; WC, waist circumference; SBP, systolic blood pressure; DBP, diastolic blood pressure; TG, triglyceride; TC, total cholesterol; HDL, high-density lipoprotein; LDL, low-density lipoprotein; and FPG, fasting plasma glucose.

### Relevance of SNPs with the risk of MS and its components

Baseline characteristics of subjects according to rs1053239 and rs2479 genotypes were presented in Supplementary Table 2 and Supplementary Table 3, respectively. For rs1053239, waist circumference was larger in CC than CG genotype, and systolic blood pressure (SBP) was higher in CG than GG genotype among MS group. As for rs2479, decreased SBP and high-density lipoprotein (HDL) were observed in AG than in GG genotype among Control group, and increased fasting glucose in AG and AA than in GG genotype among MS group, respectively.

Risk factors for MS and its components were then estimated. Genotype distributions did not significantly differ between subjects in Control and MS groups for either SNP (Supplementary Table 4). Rs2479 variation, however, was shown associated with a deteriorated fasting glucose as presented in Table 2. Individuals of AG (odds ratio 1.328 [95% confidence interval 1.066–1.655], *P* = 0.012) and AA (1.473 [1.005–2.160], *P* = 0.047) versus GG genotype were at a higher risk of elevated fasting glucose. And carriers of A allele exhibited a greater propensity for elevated fasting glucose (1.353 [1.098–1.666], *P* = 0.004). Nevertheless, neither SNP was demonstrated to be in dependent association with other MS components (Supplementary Table 4).

**Table 2:** Association analysis of rs1053239 and rs2479 with elevated fasting plasma glucose

SNP	Genotype	Normal FPG	Elevated FPG	*P* ^a^	Adjusted OR ^b^(95% CI)	*P* ^b^
		*n* (%)	*n* (%)			
rs1053239	GG	621 (37.3)	154 (30.8)	*0.027*	1.00 (ref)	-
	CG	756 (45.5)	249 (49.8)		1.015 (0.806, 1.279)	0.900
	CC	286 (17.2)	97 (19.4)		1.036 (0.768, 1.399)	0.815
	CG/CC	1042 (62.7)	346 (69.2)	*0.007*	1.021 (0.822, 1.267)	0.851
rs2479	GG	962 (57.8)	240 (48.0)	*< 0.001*	1.00 (ref)	-
	AG	582 (35.0)	213 (42.6)		1.328 (1.066, 1.655)	*0.012*
	AA	119 (7.2)	47 (9.4)		1.473 (1.005, 2.160)	*0.047*
	AG/AA	701 (42.2)	260 (52.0)	*< 0.001*	1.353 (1.098, 1.666)	*0.004*

^a^ Unadjusted *P* from univariate analysis. ^b^ Adjusted OR (95% CI) and ^b^ *P* from logistic regression analysis after adjustment for age, sex, waist circumference, blood pressure, blood lipid, smoking and alcohol intake. Statistical significance (*P* < 0.05) is indicated in italics. SNP for single nucleotide polymorphism; OR, odds ratio; and 95% CI, 95% confidence interval.

### Association of SNPs with longitudinal changes of MS components

To explore the association of SNP with the longitudinal changes of MS components, individuals free from antihypertensive, lipid-modulating, and hypoglycemic treatment over 5-year follow-up were selected. The baseline and follow-up characteristics of eligible participants were summarized in Supplementary Table 5. Average changes of MS components were stratified by rs1053239 or rs2479 genotype, respectively (Supplementary Table 6). The increase of total cholesterol was higher in CC compared with CG, and the increase of fasting glucose was lower in CG compared with GG among rs1053239 genotypes. Participants homozygous for rs2479 A allele showed a more significant increment of total cholesterol and low-density lipoprotein (LDL) than carriers of G allele.

In multivariate linear regression analysis (Table 3), C allele at rs1053239 was demonstrated to be an independent contributor to the longitudinal aggravation of SBP (β = 8.171, *P* < 0.001) and diastolic blood pressure (DBP; β = 3.708, *P* = 0.001). And A Allele at rs2479 showed an adjusted association with the exacerbation of DBP (β = 3.950, *P* = 0.003) and blood triglyceride (β = 0.296, *P* < 0.001). Besides, the female gender was indicated to be an independent determinant for an increase of waist circumference and blood triglyceride, and a decrease of HDL.

**Table 3:** Effects of rs1053239 and rs2479 variation on changes of MS components levels in participants without antihypertensive, lipid-modulating, or hypoglycemic medication treatment during 5-year follow-up

Dependent variable	Model 1					Model 2				
	Adjusted R^2^	β ^a^	*P* ^a^	β ^b^	*P* ^b^	Adjusted R^2^	β ^c^	*P* ^c^	β ^d^	*P* ^d^
ΔWC (cm)	0.593	0.021	0.985	5.514	*< 0.001*	0.595	−1.059	0.296	5.493	*< 0.001*
ΔSBP (mmHg)	0.503	8.171	*< 0.001*	1.758	0.466	0.496	4.571	0.070	0.033	0.991
ΔDBP (mmHg)	0.356	3.708	*0.001*	−1.517	0.298	0.352	3.950	*0.003*	−1.017	0.469
ΔTG (mmol/L)	0.610	0.081	0.326	0.241	*0.001*	0.499	0.296	*< 0.001*	0.431	*< 0.001*
ΔHDL (mmol/L)	0.356	−0.021	0.752	−0.166	*0.003*	0.361	0.084	0.171	−0.176	*0.002*
ΔFPG (mmol/L)	0.201	0.104	0.610	0.130	0.576	0.201	0.117	0.609	0.140	0.533

Data were calculated from multivariate linear regression analysis. In Model 1, ^a^ β and ^a^ *P* for rs1053239 variation, and ^b^ β and ^b^ *P* for sex (female vs. male), with baseline level of corresponding dependent variable and changes in levels of other metabolic syndrome components adjusted. In Model 2, ^c^ β and ^c^ *P* for rs2479 variation, and ^d^ β and ^d^ *P* for sex (female vs. male), with baseline level of corresponding dependent variable and changes in levels of other metabolic syndrome components adjusted. Statistical significance (*P* < 0.05) is indicated in italics. Adjusted R^2^ for adjusted coefficient of determination and β for partial regression coefficient.

### Effects of SNPs on efficacy of antihypertensive drugs

The influence of SNPs on blood pressure response to antihypertensive agents, including angiotensin II-targeted agents (i.e. angiotensin-converting enzyme inhibitors or angiotensin II receptor antagonists), calcium channel blockers (CCB), and diuretics, was evaluated. Characteristics of participants treated by antihypertensive monotherapy during follow-up were presented in Supplementary Table 7. Changes in blood pressure were categorized among *CIDEC* rs1053239 or rs2479 genotypes in Supplementary Table 8. Angiotensin II-targeted agents lowered blood pressure in carriers of rs1053239 C allele, or rs2479 A allele, while appeared ineffective in non-carriers. These differences were further confirmed when adjusted for other risk factors (rs1053239: β = −6.061, *P* < 0.001 for ΔSBP and β = −3.311, *P* = 0.001 for ΔDBP; rs2479: β = −8.104, *P* < 0.001 for ΔSBP and β = −5.241, *P* < 0.001 for ΔDBP; Table 4). Blood pressure in hypertensives with rs1053239 C allele was more lowered than in G allele homozygotes upon CCB intervention in univariate analysis (Supplementary Table 8). And C allele was shown to contribute to a lower efficacy of CCB in decreasing SBP after adjustment (β = 5.962, *P* = 0.003; Table 4). Diuretics functioned more intensively in rs2479 A allele carriers than non-carriers (Supplementary Table 8), which yet survived covariates correction with nominal significance (Table 4).

**Table 4:** Effects of rs1053239 and rs2479 variation on changes in blood pressure of participants treated with antihypertensive monotherapy during 5-year follow-up

Medication	Efficacy trait	Adjusted R^2 a^	β ^a^	*P* ^a^	Adjusted R^2 b^	β ^b^	*P* ^b^
Ang II-targeted agents	ΔSBP (mmHg)	0.731	−6.061	*< 0.001*	0.198	−8.104	*< 0.001*
	ΔDBP (mmHg)	0.642	−3.311	*0.001*	0.190	−5.241	*< 0.001*
CCB	ΔSBP (mmHg)	0.769	5.962	*0.003*	0.748	2.608	0.135
	ΔDBP (mmHg)	0.765	1.769	0.098	0.766	1.572	0.089
Diuretics	ΔSBP (mmHg)	0.662	1.117	0.480	0.667	−2.679	0.094
	ΔDBP (mmHg)	0.544	−0.599	0.607	0.556	−2.332	*0.048*

Data were calculated from multivariate linear regression analysis. ^a^ Adjusted R^2^, ^a^ β and ^a^ *P* for rs1053239 variation with adjustment for age, sex, smoking, alcohol intake, and baseline levels of waist circumference, blood pressure, blood lipid and fasting plasma glucose. ^b^ Adjusted R^2^, ^b^ β and ^b^ *P* for rs2479 variation with adjustment for age, sex, and baseline levels of waist circumference, blood pressure, blood lipid and fasting plasma glucose. Statistical significance (*P* < 0.05) is indicated in italics. Ang II-targeted agents for angiotensin II-targeted agents, including angiotensin-converting enzyme inhibitors and angiotensin II receptor antagonists, and CCB for calcium channel blockers.

### Influence of SNPs on cost-effectiveness of antihypertensive drugs

**Figure 1:**
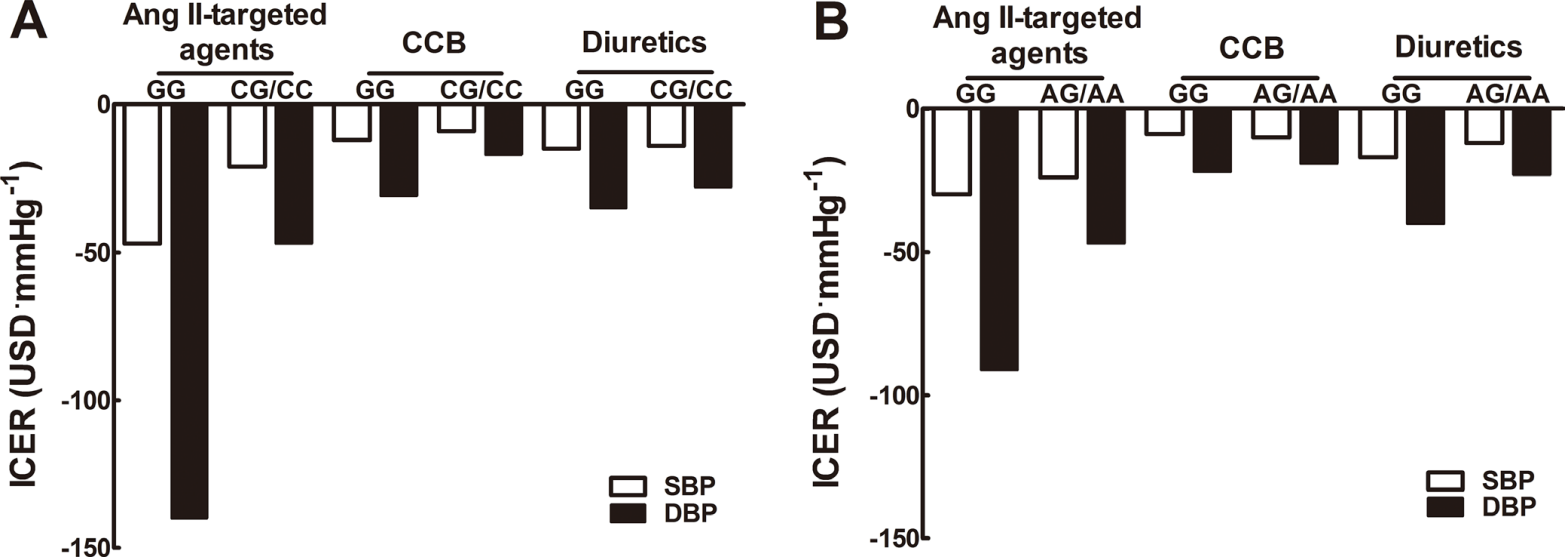
Incremental cost-effectiveness ratio of antihypertensive monotherapy Incremental cost-effectiveness ratio (USD·mmHg^−1^) of angiotensin II-targeted drugs, calcium channel blockers and diuretics was presented among *CIDEC* rs1053239 (**A**) and rs2479 (**B**) genotypes. Incremental cost-effectiveness ratio of antihypertensive drugs for each genotype was calculated as indicated in Statistical analysis.

In order to weigh the balance of drug costs and benefits, effects of SNPs on incremental cost-effectiveness ratio (ICER) of antihypertensive drugs were further assessed. For rs1053239, ICER was markedly lower for carriers of C allele versus non-carriers upon angiotensin II-targeted agents (−21 vs. -47USD·mmHg^−1^ for SBP; -47 vs. -140USD·mmHg^−1^ for DBP), and upon CCB (−17 vs. -31USD·mmHg^−1^ for DBP; USD for US dollars), while comparable upon diuretics (Figure 1A). In respect to rs2479, ICER for carriers of A allele was lower for DBP when intervened with angiotensin II-targeted drugs (−47 vs. -91USD·mmHg^−1^) or diuretics (−23 vs. -40USD·mmHg^−1^), and comparative upon CCB (Figure 1B).

## DISCUSSION

In this study, we demonstrated in a Chinese Han cohort that the minor allele of SNP rs2479 (A allele) in *CIDEC* was associated with the risk of elevated fasting plasma glucose and blood triglyceride. Both the minor allele of rs1053239 (C allele) and rs2479 in *CIDEC* predicted a longitudinal deterioration of blood pressure, and high efficacy and cost-effectiveness for angiotensin II-targeted antihypertensive drugs. Our study facilitated the identification of individuals at higher risk for MS components, and provided evidence for genotype-guided application of angiotensin II-targeted therapy.

The two SNPs in the study are both located within 3′ untranslational regions (3′ UTRs) of the *CIDEC* gene. It has been widely considered that 3′ UTRs supply platforms for assembly of key elements in the 3′ end processing, thus modulating mRNA stability and translatability, and ultimately affecting protein expression [19]. Substitution of the specific nucleotide at rs1053239 or rs2479, or particular loci under tight linkage equilibrium with these SNPs, may interfere with 3′ UTRs function and perturb the normal expression of *CIDEC*.

The risk of elevated fasting glucose was increased in carriers of A allele at rs2479 (Table 2), among whom *CIDEC* expression might be impeded in the consequence of the variation. Negative correlation was observed between fasting glucose and *CIDEC* expression in white adipose tissue [13], and blood glucose was noticeably raised due to *CIDEC* nonsense mutation [15], both in line with our findings. Follow-up study proposed A allele of rs2479 as an indicator of rapid exacerbation of blood triglyceride (Table 3). Consistently, prominent hypertriglyceridemia was elicited by loss of the C-terminal portion of *CIDEC* [15]. *CIDEC* promotes adipogenesis [6] and lipid droplets fusion [20–22], and suppresses lipolysis by inhibiting the expression [7], localization [8], and activity [9] of adipose triglyceride lipase. Therefore, *CIDEC* is essential for the maintenance of appropriate adipose mass and adequate lipid storage capacity. Mutations at rs2479 which overlaps both of the two dominate transcripts (Transcript ID: ENST00000423850, ENST00000336832; the Ensembl Database) in adipose tissue may repress *CIDEC* expression and impair lipid storage capacity in adipose tissue. As a consequence, excessive free fatty acids (FFAs) and unbalanced cluster of adipokines would be released into circulation. Indeed, *CIDEC* expression in white adipose tissue was reported to be negatively correlated with blood FFAs concentration [12]. Excessive FFAs [23] and dysregulated adipokines network [24] disrupt glucose and lipid homeostasis by triggering and accelerating systemic lipotoxicity and insulin resistance, which may be possible mechanisms of the elevation of blood glucose and triglyceride (Supplementary Figure 1).

Intriguingly, we found that the respective minor allele of rs1053239 and rs2479 independently contributed to the aggravation of blood pressure (Table 3). To date, little human data are available on the relation of *CIDEC* with blood pressure, except for the development of hypertension in a lipodystrophic patient with *CIDEC* aberrant truncation [15]. Adipokines disturbance and lipotoxicity disrupt blood pressure homeostasis by accelerating insulin resistance and sequentially reducing nitric oxide bioavailability [24]. Lipotoxicity *per se* may also facilitate the development of hypertension via activating renin-angiotensin-aldosterone system (RAAS) [25] and amplifying vasoconstrictor response to angiotensin II [26] (Supplementary Figure 1). Furthermore, higher efficacy (Table 4) of angiotensin II-targeted agents when applied to carriers of the minor allele of rs1053239 or rs2479 highlighted the contribution of RAAS activation upon *CIDEC* variation in the pathophysiology of hypertension. This study therefore suggested a role of *CIDEC* in blood pressure regulation through modulating RAAS activity, and presented positive evidence for preferential application of angiotensin II-targeted drugs for hypertensives carrying the minor allele of rs1053239 or rs2479.

Some limitations in this study need to be considered. First, the hypothetical molecular mechanism how 3′ UTR variation in *CIDEC* affected fasting glucose, blood triglyceride and blood pressure (Supplementary Figure 1) was not addressed in the present study. Second, further validation for the predictive value of the two SNPs as risk factor of MS components and pharmacogenetic indicator of angiotensin II-targeted agents is required in independent cohorts.

In conclusion, this study has indicated that 3′ UTR variation in *CIDEC* is associated with the risk of elevated fasting glucose, the progression of hypertriglyceridemia and hypertension, and the efficacy of angiotensin II-targeted antihypertensive agents. These results may shed light on the risk stratification for elevated fasting glucose, hypertriglyceridemia and hypertension, and possible approaches to genotype-guided individualized utilization of angiotensin II-targeted medication.

## MATERIALS AND METHODS

### Ethics statement

This study was approved by the ethics committee of Qilu Hospital of Shandong University. Written informed consent was obtained from all subjects.

### Study population

**Figure 2:**
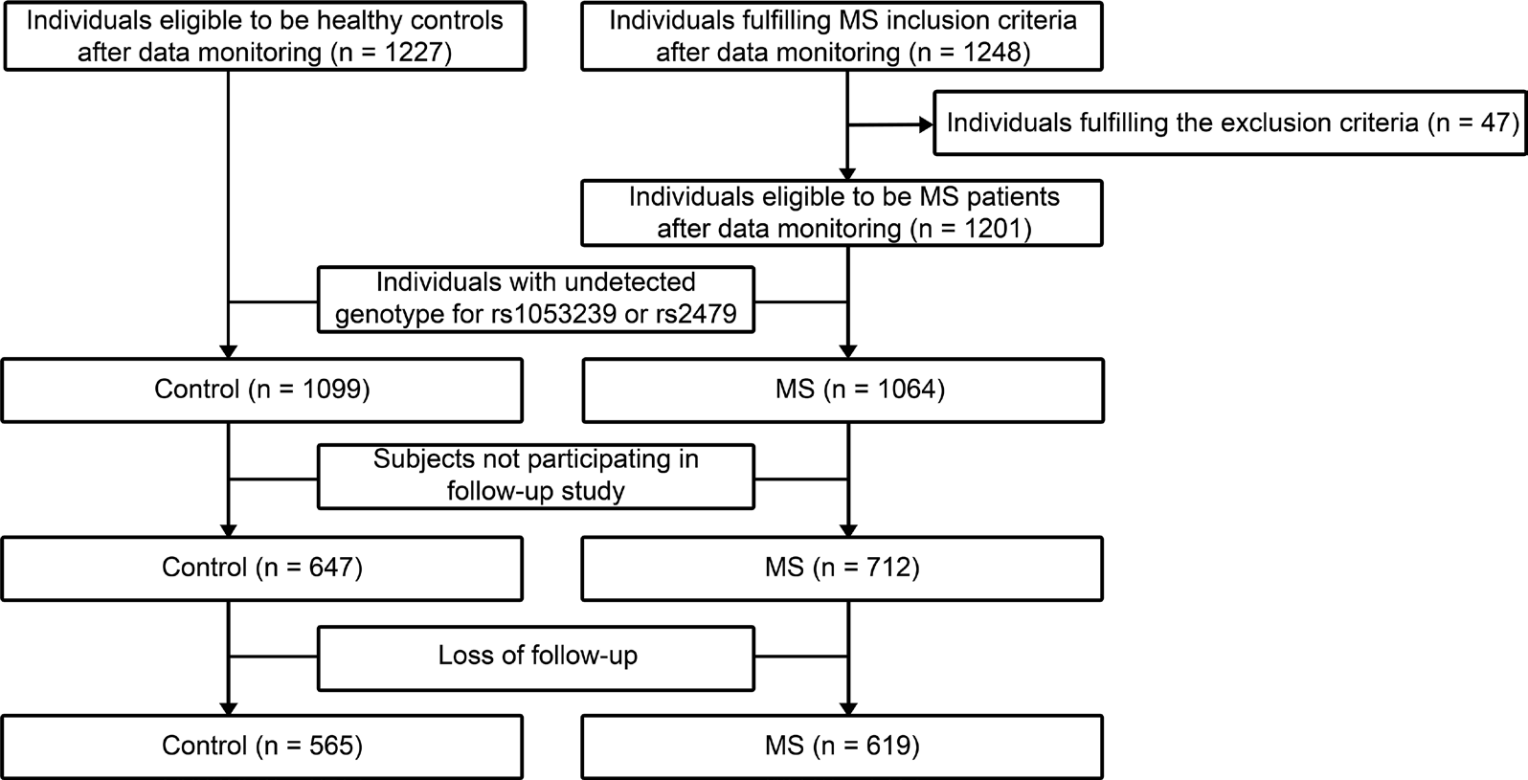
Schematic of study design

Participants in this study were of Chinese Han nationality recruited from Shandong Province from January to December of 2007. Questionnaires were conducted and blood samples were obtained. MS was defined according to the joint recommendations of the International Diabetes Federation, American Heart Association, and National Heart, Lung, and Blood Institute [27]. The exclusive criteria were: secondary hypertension, severe heart failure, renal failure or valvular heart disease. Individuals with missing covariates, missing biochemical data, undetected or discordant genotype were also excluded. A total of 1064 unrelated subjects with MS and 1099 unrelated healthy controls were enrolled. Among them, 1359 subjects participated in the follow-up study, and 1184 remained available at the end of 5-year follow-up in 2012. Details of study design were illustrated in Figure 2.

### Demographic data collection, clinical and biological assessment

Face-to-face questionnaire, physical examination and blood sample collection were conducted using a standard protocol by trained examiners at both baseline and follow-up visit. Detailed information regarding smoking and drinking habits, and medication use were included in the questionnaire. Medication use included the generic and brand name, dose, frequency and duration.

Height, weight and waist circumference were measured and body mass index was calculated. Blood pressure was measured on the right arm after a 5 minute-rest in a seated position using an OMRON HEM-7011 electronic sphygmomanometer (Omron, Dalian, China), and the average of 3 consecutive readings in a single visit was used in data analysis. Venous blood samples were obtained after an over-night fasting for laboratory estimation and DNA extraction. Plasma glucose and serum lipid concentrations were determined with a Beckman Coulter LX20 chemistry analyzer (Beckman Coulter, Brea, CA).

### SNP selection and genotyping

SNPs were selected based on the following criteria. Firstly, SNPs located within *CIDEC* gene with a MAF of > 5% in CHB according to the NCBI HapMap Database (http://hapmap.ncbi.nlm.nih.gov/). Secondly, selected SNPs were entered into Haploview Ver. 4.2 software [28] to obtain tag SNPs. Thirdly, intron variants among tag SNPs were excluded. And lastly, so far no studies have addressed the distribution regularities of the SNPs, or their relationship with MS, in Chinese population. And two SNPs rs1053239 and rs2479 were finally selected and genotyped.

Genomic DNAs were extracted from blood by Magen blood DNA kit D3133-03 (Magen, Guangzhou, China) following the manufacturer's protocols. SNPs at rs1053239 and rs2479 were genotyped in BGI, Shenzhen, China by Sequenom MassArray system (Sequenom, San Diego, CA). The PCR reaction was conducted using GeneAmp PCR System 9700 (ABI, Foster City, CA, USA). And mass determination was performed with matrix-assisted laser desorption ionization time-of-flight (MALDI-TOF) mass spectrometry [29]. Data were collected by Spectro TYPER Ver. 4.0 software (Sequenom, San Diego, CA). Call rates of genotyping were > 95% for both SNPs. A total of 120 (5%) samples were randomly selected for the concordance test, and the concordance rates were > 99% for both SNPs.

### Costs estimates

Official prices for drugs were obtained from Shandong Provincial Bureau of Pricing. Drug costs were inflated from the year of consumption to 2012 based on the inflation rate of China provided by Trading Economics. Drug costs were in US dollars (USD) according to the average exchange rate of 2012 published by the Bank of China (1USD = 6.313RMB).

### Statistical analysis

Hardy-Weinberg equilibrium for rs1053239 and rs2479 was tested using χ^2^ goodness-of-fit test. Continuous variables were presented as mean and SD or SEM, and compared by Student's *t* test, paired *t* test or analysis of variance (ANOVA) with post hoc least-significant differences *t* test. Categorical variables were presented as percentages, and compared by χ^2^ test with Bonferroni correction when appropriate. Multivariate logistic regression was performed to evaluate the risk factors of MS and its components. Longitudinal change (referred to as Δ) of MS components was calculated as the value at the end of 5-year follow-up minus that at baseline. Multiple stepwise linear regression was used to assess the contribution of SNPs to the longitudinal changes of MS components and the efficacy of antihypertensive drugs. Statistical analysis was conducted with SPSS Ver. 17.0 (SPSS, Chicago, IL). *P* values were two-tailed and considered significant when less than 0.05.

ICER of antihypertensive drugs for each genotype was calculated as follows: ICER = C / (BP_1_ - BP_0_), where BP_0_ as the blood pressure of subjects with the genotype untreated by antihypertensive drugs, and C and BP_1_ as the costs of antihypertensive drugs and blood pressure of subjects with the genotype treated by one particular antihypertensive monotherapy.

Power and Sample Size Calculation Ver. 3.1.2 software was used for power calculation [30]. The sample size of 2163 participants in the case-control study provided > 95% power to detect a 5% difference in fasting glucose between groups, with the MAF of 0.252 (CHB, the Ensembl Database). The minimum sample size of 109 participants in pharmacogenetic analysis provided > 95% power to detect a 5% difference in blood pressure between genotypes, assuming an SD of 35%∼65% of average changes in blood pressure.

## SUPPLEMENTARY MATERIALS TABLES AND FIGURES


