# Supplementary material for: Single nucleotide polymorphisms in CIDEC gene are associated with metabolic syndrome components risks and antihypertensive drug efficacy
Source: Oncotarget. 2017 Mar 10;8(16):27481–8. doi: 10.18632/oncotarget.16078 (PMC5432350; doi:10.18632/oncotarget.16078)
Supplement: Supplementary file 1 [file oncotarget-08-27481-s001.pdf]

Single nucleotide polymorphisms in *CIDEC* gene are associated with metabolic syndrome components risks and antihypertensive drug efficacy

Supplementary Materials

Supplementary Table 1: Hardy-Weinberg equilibrium test for CIDEC rs1053239 and rs2479 genotype distribution

		rs1053239				rs2479			
	n	GG	CG	CC	*P* [a]	GG	AG	AA	*P* [b]
		OF (EF)	OF (EF)	OF (EF)		OF (EF)	OF (EF)	OF (EF)	
Control	1099	404 (388.59)	499 (529.82)	196 (180.59)	0.178	611 (598.47)	400 (425.06)	88 (75.47)	0.164
MS	1064	371 (365.95)	506 (516.09)	187 (181.95)	0.655	591 (584.33)	395 (408.33)	78 (71.33)	0.446
Total	2163	775 (754.51)	1005 (1045.98)	383 (362.51)	0.204	1202 (1182.80)	795 (833.40)	166 (146.80)	0.139

[a] *P* for rs1053239 variation, and [b] *P* for rs2479 variation. MS for metabolic syndrome; OF, observed frequency; and EF, expected frequency.

Supplementary Table 2: Baseline clinical and biological characteristics of participants among *CIDEC* rs1053239 genotypes

	Control (*n* = 1099)			MS (*n* = 1064)		
	GG	CG	CC	GG	CG	CC
n	404	499	196	371	506	187
Male (%)	43.3	43.3	42.3	40.2	38.9	47.1
Age (years)	49.29 ± 12.05	50.08 ± 11.26	48.86 ± 11.67	49.35 ± 8.52	50.39 ± 8.64	49.24 ± 8.99
BMI (kg/m^2)	21.97 ± 2.63	21.99 ± 2.51	21.98 ± 2.46	27.41 ± 3.07	27.25 ± 3.21	27.77 ± 3.04
WC (cm)	74.95 ± 5.99	74.81 ± 6.23	75.18 ± 6.20	90.99 ± 7.36	90.38 ± 7.90	92.20 ± 7.71[b]
SBP (mmHg)	116.18 ± 8.30	115.64 ± 8.42	115.18 ± 8.62	146.04 ± 17.16	150.15 ± 17.50[a]	148.96 ± 19.53
DBP (mmHg)	73.76 ± 5.78	73.19 ± 6.16	74.12 ± 6.59	90.25 ± 9.10	90.54 ± 9.66	91.65 ± 10.37
TG (mmol/L)	0.90 ± 0.30	0.93 ± 0.31	0.91 ± 0.35	2.11 ± 1.02	2.13 ± 1.05	2.16 ± 1.18
TC (mmol/L)	4.12 ± 0.53	4.11 ± 0.55	4.07 ± 0.57	4.59 ± 1.03	4.60 ± 0.99	4.60 ± 1.10
HDL (mmol/L)	1.58 ± 0.31	1.59 ± 0.34	1.53 ± 0.31	1.46 ± 0.45	1.46 ± 0.44	1.48 ± 0.43
LDL (mmol/L)	2.53 ± 0.52	2.52 ± 0.55	2.54 ± 0.55	3.14 ± 0.74	3.13 ± 0.70	3.11 ± 0.81
FPG (mmol/L)	4.54 ± 0.51	4.57 ± 0.51	4.51 ± 0.45	5.66 ± 1.58	5.80 ± 1.68	5.89 ± 1.46

Data are mean ± SD for continuous variables, or proportions for categorical variable. In MS group, [a] $P < 0.01$ vs. GG; [b] $P < 0.01$ vs. CG. BMI for body mass index; WC, waist circumference; SBP, systolic blood pressure; DBP, diastolic blood pressure; TG, triglyceride; TC, total cholesterol; HDL, high-density lipoprotein; LDL, low-density lipoprotein; and FPG, fasting plasma glucose.

Supplementary Table 3: Baseline clinical and biological characteristics of participants among *CIDEC* rs2479 genotypes

	Control (n = 1099)			MS (n = 1064)		
	GG	AG	AA	GG	AG	AA
n	611	400	88	591	395	78
Male (%)	42.9	43.8	42.0	40.9	40.0	43.6
Age (years)	49.39 ± 11.88	50.20 ± 11.49	48.00 ± 10.37	49.63 ± 8.70	50.33 ± 8.69	48.73 ± 8.26
BMI (kg/m^2)	22.01 ± 2.59	21.92 ± 2.50	22.10 ± 2.44	27.33 ± 3.08	27.44 ± 3.29	27.66 ± 2.70
WC (cm)	74.89 ± 5.99	74.86 ± 6.34	75.52 ± 6.22	90.73 ± 7.49	91.06 ± 8.10	91.55 ± 7.22
SBP (mmHg)	116.24 ± 8.22	114.90 ± 8.55 [a]	116.28 ± 8.90	147.52 ± 17.73	149.79 ± 17.21	149.54 ± 21.19
DBP (mmHg)	73.61 ± 5.97	73.28 ± 6.22	74.51 ± 6.53	90.44 ± 9.48	90.98 ± 9.62	90.38 ± 10.50
TG (mmol/L)	0.90 ± 0.30	0.92 ± 0.31	0.97 ± 0.37	2.17 ± 1.06	2.11 ± 1.10	1.89 ± 0.92
TC (mmol/L)	4.12 ± 0.56	4.09 ± 0.54	4.11 ± 0.52	4.57 ± 1.02	4.64 ± 1.01	4.58 ± 1.18
HDL (mmol/L)	1.60 ± 0.33	1.55 ± 0.32[a]	1.55 ± 0.29	1.46 ± 0.45	1.48 ± 0.43	1.46 ± 0.46
LDL (mmol/L)	2.52 ± 0.55	2.55 ± 0.52	2.56 ± 0.50	3.11 ± 0.74	3.17 ± 0.70	3.09 ± 0.85
FPG (mmol/L)	4.55 ± 0.52	4.54 ± 0.49	4.53 ± 0.44	5.61 ± 1.57	5.92 ± 1.68[b]	6.20 ± 1.41[b]

Data are mean ± SD for continuous variables, or proportions for categorical variable. In Control group, [a] $P < 0.05$ vs. GG. In MS group, [b] $P < 0.01$ vs. GG.

Supplementary Table 4: Association analysis of rs1053239 and rs2479 with MS and its components

Phenotype	SNP	Genotype	Control	Case	P^a	Adjusted OR (95% CI) [b]	P^b
			n (%)	*n* (%)			
MS	rs1053239	GG	404 (36.8)	371 (34.9)	0.577	1.00 (ref)	-
		CG	499 (45.4)	506 (47.6)		1.018 (0.843, 1.229)	0.852
		CC	196 (17.8)	187 (17.6)		0.945 (0.739, 1.207)	0.649
	rs2479	GG	611 (55.6)	591 (55.5)	0.819	1.00 (ref)	-
		AG	400 (36.4)	395 (37.1)		0.972 (0.811, 1.165)	0.758
		AA	88 (8.0)	78 (7.3)		0.875 (0.630, 1.215)	0.426
Elevated WC	rs1053239	GG	442 (36.6)	333 (34.9)	0.617	1.00 (ref)	-
		CG	559 (46.3)	446 (46.7)		0.934 (0.773, 1.128)	0.477
		CC	207 (17.1)	176 (18.4)		0.989 (0.774, 1.264)	0.929
	rs2479	GG	678 (56.1)	524 (54.9)	0.814	1.00 (ref)	-
		AG	440 (36.4)	355 (37.2)		0.967 (0.806, 1.160)	0.719
		AA	90 (7.5)	76 (8.0)		1.008 (0.726, 1.399)	0.964
Elevated blood pressure	rs1053239	GG	418 (36.7)	357 (34.8)	0.533	1.00 (ref)	-
		CG	516 (45.3)	489 (47.7)		0.994 (0.824, 1.199)	0.950
		CC	204 (17.9)	179 (17.5)		0.906 (0.709, 1.158)	0.432
	rs2479	GG	635 (55.8)	567 (55.3)	0.665	1.00 (ref)	-
		AG	411 (36.1)	384 (37.5)		0.979 (0.817, 1.173)	0.815
		AA	92 (8.1)	74 (7.2)		0.842 (0.606, 1.170)	0.306
Elevated triglyceride	rs1053239	GG	531 (36.1)	244 (35.2)	0.918	1.00 (ref)	-
		CG	680 (46.3)	325 (46.9)		0.986 (0.807, 1.205)	0.891
		CC	259 (17.6)	124 (17.9)		0.951 (0.733, 1.235)	0.708
	rs2479	GG	801 (54.5)	401 (57.9)	0.277	1.00 (ref)	-
		AG	550 (37.4)	245 (35.4)		0.860 (0.709, 1.045)	0.129
		AA	119 (8.1)	47 (6.8)		0.750 (0.523, 1.076)	0.118
Reduced HDL	rs1053239	GG	649 (35.7)	126 (36.6)	0.660	1.00 (ref)	-
		CG	842 (46.3)	163 (47.4)		0.916 (0.713, 1.177)	0.491
		CC	328 (18.0)	55 (16.0)		0.758 (0.540, 1.066)	0.111
	rs2479	GG	1001 (55.0)	201 (58.4)	0.378	1.00 (ref)	-
		AG	680 (37.4)	115 (33.4)		0.795 (0.619, 1.020)	0.071
		AA	138 (7.6)	28 (8.1)		0.906 (0.585, 1.403)	0.659

[a]Unadjusted *P* from univariate analysis; [b] Adjusted OR (95% CI) and [b] *P* from logistic regression analysis after adjustment for sex, age, smoking and alcohol intake. SNP for single nucleotide polymorphism; OR, odds ratio; and 95% CI, 95% confidence interval.

Supplementary Table 5: Clinical and biological characteristics of participants without antihypertensive, lipid-modulating, or hypoglycemic medication treatment at baseline and at the end of 5-year follow-up

	Baseline (2007)	Follow-up (2012)
BMI (kg/m^2)	22.71 ± 3.11	24.65 ± 4.03 [a]
WC (cm)	76.52 ± 8.00	81.48 ± 7.18 [a]
SBP (mmHg)	117.56 ± 13.84	132.79 ± 17.11 [a]
DBP (mmHg)	75.20 ± 9.05	81.02 ± 9.15 [a]
TG (mmol/L)	1.08 ± 0.66	1.38 ± 0.60 [a]
TC (mmol/L)	4.12 ± 0.67	4.31 ± 0.99 [b]
HDL (mmol/L)	1.56 ± 0.35	1.26 ± 0.36 [a]
LDL (mmol/L)	2.57 ± 0.61	2.42 ± 0.95 [b]
FPG (mmol/L)	4.64 ± 0.98	5.46 ± 1.85 [a]

Data are mean ± SD (n = 238; male (%): 43.3; age in 2007: 48.41). [a] $P < 0.001$ and [b] $P < 0.05$ vs. Baseline (2007).

Supplementary Table 6: Changes of clinical and biological characteristics of participants without antihypertensive, lipid-modulating, or hypoglycemic medication treatment among *CIDEC* rs1053239 and rs2479 genotypes

	rs1053239			rs2479		
	GG	CG	CC	GG	AG	AA
n	78	115	45	124	95	19
Male (%)	37.2	45.2	48.9	43.5	37.9	68.4 [c]
Age (years)	50.46 ± 1.20	52.69 ± 0.99	51.84 ± 1.64	51.70 ± 0.97	51.67 ± 1.15	53.05 ± 1.57
ΔBMI (kg/m^2)	1.50 ± 0.63	2.10 ± 0.48	2.26 ± 0.70	1.25 ± 0.48	2.90 ± 0.51	1.58 ± 1.16
ΔWC (cm)	5.43 ± 1.34	4.89 ± 1.00	4.32 ± 1.66	4.90 ± 1.01	5.37 ± 1.13	3.28 ± 2.65
ΔSBP (mmHg)	14.31 ± 2.36	14.47 ± 2.28	18.78 ± 3.31	13.83 ± 2.04	16.67 ± 2.43	17.15 ± 4.71
ΔDBP (mmHg)	4.72 ± 1.43	5.45 ± 1.27	8.64 ± 2.08	4.49 ± 1.20	6.84 ± 1.40	9.31 ± 2.81
ΔTG (mmol/L)	0.34 ± 0.08	0.20 ± 0.09	0.47 ± 0.13	0.25 ± 0.08	0.37 ± 0.11	0.29 ± 0.12
ΔTC (mmol/L)	0.10 ± 0.15	0.08 ± 0.09	0.61 ± 0.19 [b]	0.07 ± 0.11	0.19 ± 0.11	0.94 ± 0.27 [d e]
ΔHDL (mmol/L)	−0.26 ± 0.06	−0.32 ± 0.05	−0.33 ± 0.06	−0.33 ± 0.05	−0.26 ± 0.05	−0.32 ± 0.10
ΔLDL (mmol/L)	−0.27 ± 0.13	−0.21 ± 0.10	0.21 ± 0.18	−0.18 ± 0.11	−0.27 ± 0.11	0.65 ± 0.26 [d e]
ΔFPG (mmol/L)	1.16 ± 0.28	0.46 ± 0.17 [a]	1.17 ± 0.31	0.90 ± 0.20	0.61 ± 0.19	1.41 ± 0.62

Data are mean ± SEM for continuous variables, or proportions for categorical variable (n = 238). For rs1053239, [a] $P < 0.05$ vs. GG; [b] $P < 0.05$ vs. CG. For rs2479, [c] $P < 0.05$ vs. AG; [d] $P < 0.01$ vs. GG and [e] $P < 0.01$ vs. AG.

Supplementary Table 7: Clinical and biological characteristics of participants treated with antihypertensive monotherapy at baseline and at the end of 5-year follow-up

	Baseline (2007)	Follow-up (2012)
BMI (kg/m^2)	26.28 ± 3.73	26.69 ± 4.59
WC (cm)	87.38 ± 9.42	86.79 ± 7.16
SBP (mmHg)	147.62 ± 21.97	142.16 ± 7.41 [a]
DBP (mmHg)	87.79 ± 11.40	84.14 ± 6.27 [a]
TG (mmol/L)	1.95 ± 1.44	1.45 ± 0.61 [a]
TC (mmol/L)	4.66 ± 1.10	4.57 ± 1.15
HDL (mmol/L)	1.57 ± 0.49	1.26 ± 0.36 [a]
LDL (mmol/L)	3.09 ± 0.77	2.77 ± 0.98 [a]
FPG (mmol/L)	5.74 ± 2.00	5.79 ± 2.16

Data are mean ± SD (*n* = 362; male (%): 27.1; age in 2007: 53.35). [a] $P < 0.001$ vs. Baseline (2007).

Supplementary Table 8: Changes of blood pressure of participants treated with antihypertensive monotherapy among *CIDEC* rs1053239 and rs2479 genotypes

Medication	Efficacy trait	rs1053239		rs2479	
		GG	CG/CC	GG	AG/AA
Ang II-targeted	*n*	38	71	58	51
agents (*n* = 109)	ΔSBP (mmHg)	4.68 ± 1.19	−8.37 ± 1.31 [a]	−0.05 ± 1.45	−8.10 ± 1.53 [c]
	ΔDBP (mmHg)	1.91 ± 0.85	−4.55 ± 0.95 [a]	0.29 ± 0.95	−5.24 ± 1.02 [c]
CCB	*n*	35	79	65	49
(*n* = 114)	ΔSBP (mmHg)	−2.29 ± 2.34	−8.54 ± 2.02 [b]	−8.46 ± 2.33	−4.18 ± 2.00
	ΔDBP (mmHg)	−1.35 ± 1.73	−5.66 ± 0.87 [b]	−4.83 ± 1.16	−3.68 ± 1.13
Diuretics	*n*	58	81	85	54
(*n* = 139)	ΔSBP (mmHg)	−5.55 ± 1.96	−5.98 ± 1.60	−3.53 ± 1.51	−9.37 ± 2.04 [d]
	ΔDBP (mmHg)	−3.33 ± 0.82	−4.72 ± 1.19	−2.69 ± 0.86	−6.43 ± 1.41 [d]

Participants were treated with monotherapy of antihypertensive drugs, i.e. Ang II-targeted agents, CCB, or diuretics over 5-year follow-up. Data are mean ± SEM for continuous variables. For rs1053239, [a] $P < 0.001$ and [b] $P < 0.05$ vs. GG. For rs2479, [c] $P < 0.001$ and [d] $P < 0.05$ vs. GG. Ang II-targeted agents for angiotensin II-targeted agents, including angiotensin-converting enzyme inhibitors and angiotensin II receptor antagonists, and CCB for calcium channel blockers.

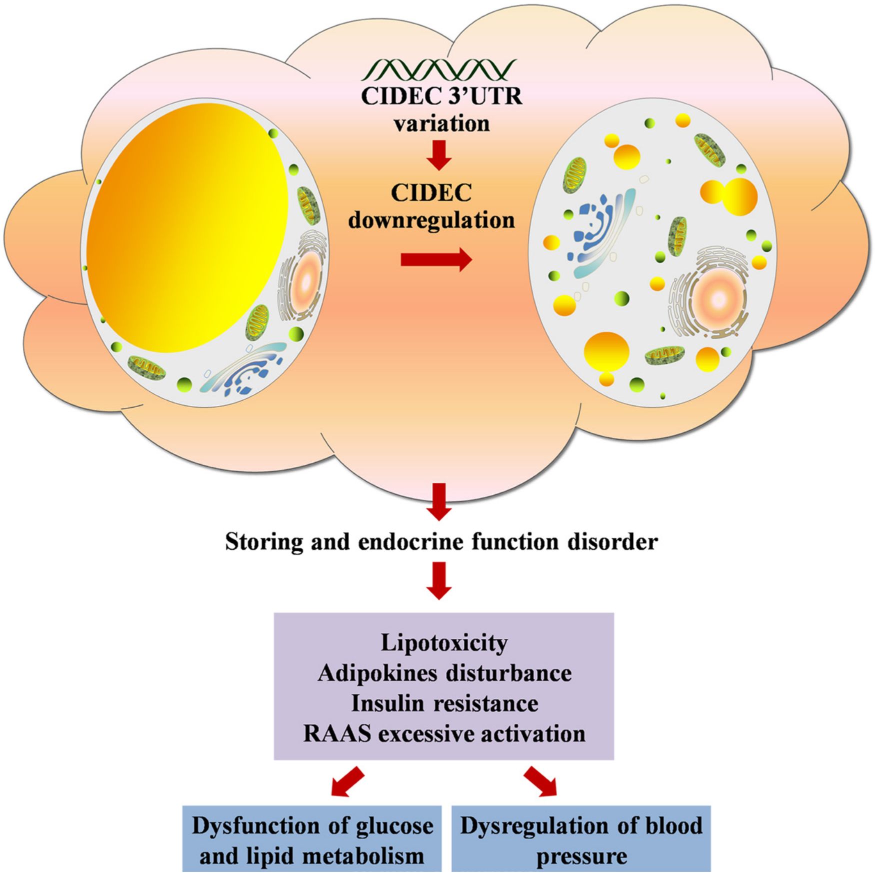

Supplementary Figure 1: Schematic illustration of the hypothetical mechanism how 3′ UTR variation in *CIDEC* affected fasting glucose, blood triglyceride and blood pressure. Nucleotide substitution at the 3′ UTR variation in *CIDEC* gene might interfere with 3′ UTRs function and disturb the normal expression of *CIDEC*. As a result, lipid droplets would be diminished and lipid mobilization exacerbated, leading to impaired lipid storage capacity and disordered endocrine function of adipose tissue. The excessive FFAs and unbalanced cluster of adipokines released from adipose tissue would trigger insulin resistance and accelerate RAAS activation, and in consequence disturb glucose and lipid metabolism and blood pressure regulation.
